# Selectively false-positive radionuclide scan in a patient with sarcoidosis and papillary thyroid cancer: a case report and review of the literature

**DOI:** 10.1186/s40463-015-0069-3

**Published:** 2015-05-15

**Authors:** Nicole L Lebo, Francois Raymond, Michael J Odell

**Affiliations:** Department of Otolaryngology-Head and Neck Surgery, The Ottawa Hospital, University of Ottawa, 501 Smyth Rd., Box 216, Ottawa, ON K1H 8L6 Canada; Department of Medicine, Division of Nuclear Medicine, University of Ottawa, Ottawa, Canada

**Keywords:** Thyroid gland, Thyroid neoplasms, Nuclear medicine, Sarcoidosis

## Abstract

**Background:**

Radioiodine and Tc-99 m pertechnetate scans are routinely relied upon to detect metastasis in papillary thyroid cancer; false-positive scans are relatively rare. To our knowledge, no published reports exist of sarcoidosis causing such selectively false-positive scans.

**Methods:**

We present a case of a 41-year-old woman with known metastatic papillary thyroid cancer (T1bN1aMx) in whom sarcoidosis-affected cervical and mediastinal lymph nodes demonstrated uptake of thyroid-targeting radionuclides. Only the minority of these nodes demonstrated radionuclide uptake, raising the suspicion of adjacent or coexisting sarcoid and metastatic involvement. Selective uptake of thyroid-targeted radionuclides by isolated sarcoidosis is, to our knowledge, a previously undocumented occurrence.

**Results:**

Biopsies of uptake-negative mediastinal nodes revealed sarcoidosis. Pathology from a subsequent neck dissection excising uptake-positive cervical nodes also showed sarcoidosis, with no coinciding malignancy.

**Conclusions:**

We document a case of sarcoidosis causing a selectively false-positive thyroid scintigraphy scan. It is useful for clinicians to be aware of potential false-positives and deceptive patterns on radionuclide scans when managing patients with both well-differentiated thyroid cancer and a co-existing disease affecting the nodal basins draining the thyroid gland.

## Background

Papillary thyroid cancer is a relatively common malignancy, occurring with markedly increasing incidence in developed countries [1]. The approach to treatment has been fairly consistent for decades; in the majority of cases, the paradigm continues to be surgery followed by radioactive iodine treatment of residual or metastatic tissue [2]. Radioiodine (particularly ^131^I) scans are routinely relied upon to detect metastasis because of their perceived specificity in identifying normal and malignant thyroid tissue. Detection hinges on retention of the ability to concentrate and store iodine by well-differentiated thyroid malignancies [3].

In current practice, Tc-99 m pertechnetate thyroid scans (localized to the head and neck) are often used in place of radioiodine scans for identifying functional thyroid tissue, with only occasional result discrepancies [4]. Pertechnetate is trapped by the same sodium-iodine symporter as radioiodine, but is washed out relatively rapidly as it is not stored by follicular cells [5]. Pertechnetate thyroid scans carry the advantages of lower radiation doses, faster results, and greater accessibility compared to ^131^I [6]. ^131^I is the preferred radionuclide for whole body imaging, where a longer half-life is desirable [7].

False-positive radioiodine scans are rare. Numerous artifacts and physiologic variants are known to consistently take up radioiodine but are easily discounted by experienced nuclear medicine physicians. Several non-thyroidal disease processes have been noted to demonstrate uptake in less predictable patterns. These include non-thyroidal neoplasms, serous cysts, and focal fungal infections [3,8]. False-positive pertechnetate thyroid scans are also uncommon. As the pertechnetate ion behaves similar to iodide, it displays analogous physiologic and artifactual uptake patterns (ex. by salivary glands, gastric mucosa) [9].

Sarcoidosis is a systemic disorder of unknown etiology, characterized by development of non-caseating granulomas in affected organs. Lung and thoracic lymph node involvement are most common, though many other sites can be involved. Clinical presentation is not uncommonly an incidental finding on imaging conducted for other purposes. Sarcoidosis exhibits a predilection for adults under 40 and specific racial groups, particularly those of African descent [10]. To our knowledge, sarcoidosis has not previously been described in the literature as a disease entity demonstrating conclusive radioiodine or pertechnetate uptake. A report exists of sarcoidosis mimicking a suprasellar tumour on a pertechnetate brain scan, but the increased uptake was attributed to general disruption of the blood–brain-barrier, not to specific uptake by sarcoid tissue [11].

Here we document a case of sarcoidosis causing a false-positive radionuclide scan in a patient with papillary thyroid cancer, and review the pertinent literature on the topic.

## Case presentation

### Case details

A 41-year-old Caucasian woman was referred with a left-sided thyroid nodule. Fine-needle aspiration revealed a diagnosis of papillary thyroid carcinoma; the patient thus underwent a total thyroidectomy and left central nodal dissection. Pathology showed multifocal papillary thyroid carcinoma and one positive lymph node (AJCC Stage T1bN1aMx). In addition, epithelioid granulomas were identified within this node. This was reported as suggesting a concurrent infectious process, though the possibility of sarcoidosis was not ruled out.

In preparation for postoperative radioiodine remnant ablation, pre-ablation imaging was performed with Tc-99 m pertechnetate thyroid scanning. The patient was injected with 370 MBq (10 mCi) of Tc-99 m pertechnetate IV. Imaging began 15 minutes post-injection. Static images were acquired using a Siemens Symbia T1 gamma camera with a pinhole collimator of 4 mm aperture (matrix 128x128, zoom factor 2). A first static image was acquired over 108 seconds at 14.9 cm from the neck, with the patient supine and markers at the sternal notch and thyroid cartilage. A second static image was acquired from the same distance over 300 seconds. A third, magnified, image was obtained at a distance of 3 cm from the patient's neck.

SPECT/CT of the same area was performed over a 180° arc (clockwise rotation), using 128 projections (64 views x dual heads) at 12 s per view. Images were acquired into a 128x128 matrix at a zoom factor of 1, using a step-and-shoot rotation with an auto-continuing non-circular orbit. Scout CT was obtained (30 mA, 130 kV, scan time 0.8 s), followed by routine CT at a slice thickness of 5 mm, effective current of 17 mA, and voltage of 130 kV.

**Figure 1:**
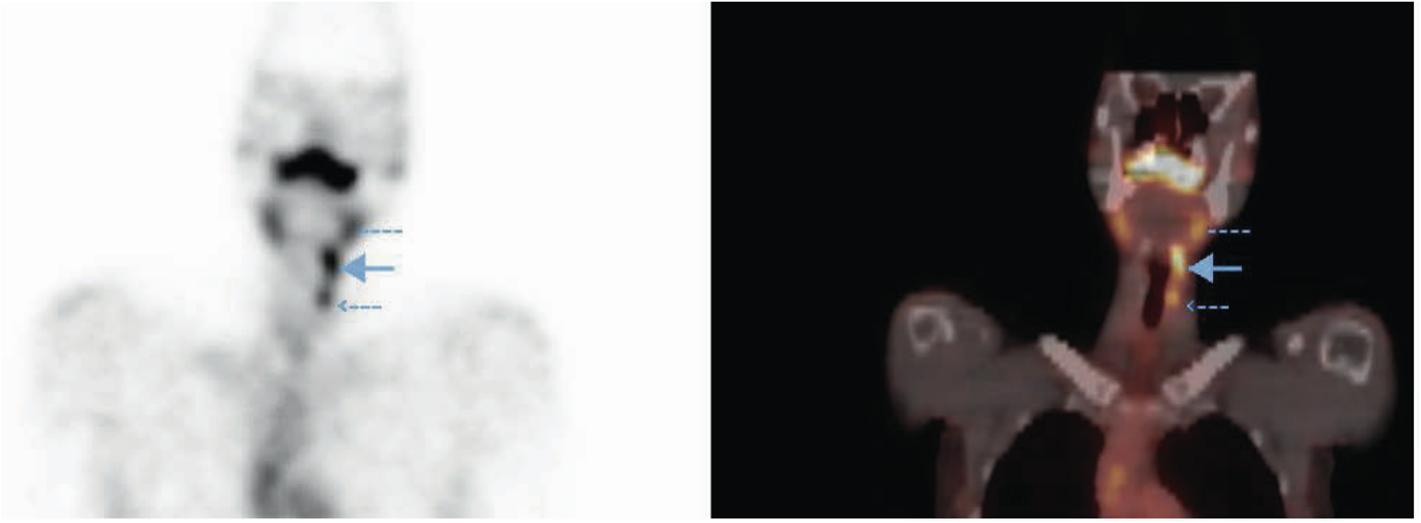
Corresponding SPECT and fused SPECT/CT images showing concerning cervical lesions. SPECT coronal slice (*left*) and fused SPECT/CT image (*right*) demonstrating abnormal foci of accumulation of ^99m^Tc0_4_^−^ radioactive tracer in a lymph node of the left jugular chain, ipsilateral to the known thyroid tumor. [Dotted line (−−-) indicates submandibular gland; small dotted arrow indicates thyroid remnant; large arrow indicates the area of concern in the lateral jugular chain]. No appreciable ^99m^Tc0_4_^−^ uptake is observed in the superior mediastinum, where extensive nodal disease was identified on plain CT-Neck.

The radionuclide imaging showed uptake-positive lesions along the left internal jugular chain, highly suggestive of metastases (Figure 1). At that time, total body ^131^I uptake was elevated at 7.4% (expected normal < 5%), and un-stimulated (TSH 10.28 mU/L) serum thyroglobulin was 5.5 pmol/L. Plain CT of the neck showed extensive nodal disease in the neck, as well as in the mediastinum. Notably, unlike the cervical nodes, these mediastinal nodes had not shown appreciable uptake on the pertechnetate thyroid scan. CT of the thorax showed mediastinal and bilateral pulmonary hilar lymphadenopathy. Percutaneous biopsies of the mediastinal and pulmonary nodes demonstrated findings consistent with a diagnosis of sarcoidosis and no coinciding malignancy.

The patient was presented with the option of fine-needle aspiration biopsy of the scan-positive cervical nodes as a means of confirming or discounting their metastatic etiology (versus being purely additional foci of sarcoidosis) before deciding whether to proceed with selective neck dissection. It was agreed that a negative FNAB would not be sufficiently reassuring to justify no further surgery, given the suspicious location, the absence of previous reports of convincing pertechnetate or radioiodine uptake by sarcoid tissue, and, moreover, the selective uptake of pertechnetate by the cervical nodes alone.

Left lateral neck dissection was therefore performed without complication. Final surgical pathology showed all level II, III, IV nodes negative for malignancy, and level IV nodes positive for granulomatous lymphadenitis compatible with sarcoidosis. The patient underwent radioiodine ablation in October 2009. Follow-up imaging (^131^I whole body scanning) to date has been normal, and there has been no clinical evidence of malignancy relapse in the primary site or the neck.

## Discussion

Papillary thyroid cancer, constituting >90% of thyroid malignancies [2], is a relatively common disease in young, Caucasian women [12]. Radioiodine and Tc-99 m pertechnetate scans are relied upon for detection of recurrence and metastasis because of their perceived specificity for normal and well-differentiated malignant thyroid tissue. ^131^I whole body scans have become the particular scan of choice in monitoring for spread and relapse [3].

Survival rates for papillary cancer are high, but follow-up including appropriate and timely management of recurrence is important to avoid uncontrolled loco-regional disease. The regional metastatic pattern for papillary cancer is fairly predictable, with initial spread to central compartment (level VI) nodes, followed typically by ipsilateral jugular nodes, with level III and IV involvement in particular being more common than level II [13,14]. Nodal involvement is frequently encountered [2], thus ipsilateral cervical nodes deserve significant attention during work-up for metastatic spread. In our patient, the identification of new lesions along the length of the internal jugular chain ipsilateral to her thyroid nodule and lone metastatic central node was highly suspicious for malignant spread. Notably, patients with sarcoidosis have been observed to develop sarcoid-granulomas in cervical nodes [10]. Had radionuclide uptake been consistent amongst the cervical, mediastinal, and pulmonary nodes (either all positive, or all negative), cervical lymphadenopathy attributable to sarcoidosis would have been a reasonable alternative diagnosis.

Ultimately, uptake of a thyroid-targeted radionuclide by our patient’s cervical lesions and not by the mediastinal nodes known to be affected by sarcoidosis was the most convincing factor supporting our anticipation of papillary cancer spread. Further, with no previous evidence of sarcoidosis causing false-positive scans, and no immediately obvious physiologic reason to expect pertechnetate or iodine uptake by sarcoid granulomas, our patient’s underlying sarcoidosis was not initially a convincing cause for her scan-positive lesions.

## Conclusions

The unexpected finding of sarcoidosis as the pathology underlying our patient’s scan-positive cervical lesions is, to our knowledge, the first report of sarcoidosis demonstrating uptake of the radionuclides used in assessing thyroid tissue. For surgeons using nuclear imaging to work-up thyroid malignancy in patients with sarcoidosis, this is an important phenomenon, demonstrating a need to be cognizant of the possibility for false-positives.

## Consent

Written informed consent was obtained from the patient for publication of this case report and any accompanying images. A copy of the written consent is available for review by the Editor-in-Chief of this journal.
